# Greater ambient air pollution exposure is associated with worse respiratory symptoms in men and women with HIV and chronic lung disease: a cohort study

**DOI:** 10.1186/s12931-025-03398-0

**Published:** 2025-12-19

**Authors:** May-Lin Wilgus, Andrew Edmonds, Kayleigh P. Keller, Jessica L. Elf, Alison G. Abraham, Surya P. Bhatt, Russell G. Buhr, Mardge H. Cohen, Roger Detels, M. Bradley Drummond, Margaret A. Fischl, Robert Foronjy, H. Dean Hosgood, Laurence Huang, Ken M. Kunisaki, Meredith McCormack, Alison Morris, Sarath Raju, Catalina Ramirez, Jacqueline Rudolph, Valentina Stosor, Donald P. Tashkin, Adam Visconti, Sheri D. Weiser, Gina Wingood, Igor Barjaktarevic

**Affiliations:** 1 University of California Los Angeles, Los Angeles, CA 90095 USA; 2 University of North Carolina at Chapel Hill, Chapel Hill, NC USA; 3 Colorado State University, Fort Collins, CO USA; 4 University of Colorado, Aurora, CO USA; 5 University of Alabama at Birmingham, Birmingham, AL USA; 6 Stroger Hospital of Cook County Health and Human Services, Chicago, IL USA; 7 University of Miami School of Medicine, Miami, FL USA; 8 SUNY Downstate Health Sciences University, Brooklyn, NY USA; 9 Albert Einstein College of Medicine, Bronx, NY USA; 10 University of California San Francisco, San Francisco, CA USA; 11 Boise VA Medical Center, Boise, ID USA; 12 Johns Hopkins University, Baltimore, MD USA; 13 University of Pittsburgh School of Medicine, Pittsburgh, PA USA; 14 Center for Advanced Biotechnology and Medicine, Rutgers Robert Wood Johnson Medical School, Piscataway, NJ USA; 15 Northwestern University Feinberg School of Medicine, Chicago, IL USA; 16 Georgetown University, Washington, DC USA; 17 Columbia University, New York, NY USA

**Keywords:** HIV, Air pollution, PM_2.5_, Ozone, COPD, Impaired DLCO, SGRQ, mMRC, MACS, WIHS, MWCCS

## Abstract

**Background:**

COPD and impairment in diffusing capacity for carbon monoxide (DLCO) are common comorbidities in people with HIV (PWH). HIV may increase susceptibility to inhaled toxins including air pollution. In PWH and people without HIV (PWoH), we investigated whether air pollution exposure was associated with within-group differences in lung function or respiratory symptoms, and whether these associations differed by HIV serostatus or the presence of underlying lung disease.

**Methods:**

We analyzed cross-sectional data from the Multicenter AIDS Cohort Study (MACS) and the Women’s Interagency HIV Study (WIHS), including participants with pulmonary function tests and accompanying standardized respiratory questionnaires in 2017–2020. The participants were linked to fine particulate matter (PM_2.5_) and ozone exposure data. Associations between exposures and respiratory outcomes were quantified with regression models. Two subgroup analyses were conducted, restricting to individuals with COPD (FEV_1_/FVC ratio < 0.7) or impaired DLCO (< 80% predicted).

**Results:**

338 MACS participants and 1073 WIHS participants were included. Overall, there were no significant associations between pollution exposures and either lung function or respiratory symptoms. In PWH with COPD, 1 µg/m^3^ greater exposure to PM_2.5_ was associated with worse St. George’s Respiratory Questionnaire (SGRQ) score (4.04 points; 95% CI 0.36–7.72) and worse modified Medical Research Council (mMRC) dyspnea score (0.28 points; 95% CI 0.02–0.53). In PWH with impaired DLCO, 1 µg/m^3^ greater PM_2.5_ exposure was associated with worse SGRQ (2.14 points, 95% CI 0.2–4.08) and mMRC (0.16 points, 95% CI 0.02–0.29). There were no significant associations between PM_2.5_ and respiratory symptoms in PWoH with COPD or impaired DLCO. Ozone exposure was not associated with respiratory symptoms in PWH or PWoH.

**Conclusions:**

PM_2.5_ exposure may act synergistically with HIV infection to worsen respiratory symptoms in people with chronic lung disease. Further study is needed to determine if air pollution leads to decline in lung function in PWH.

**Supplementary Information:**

The online version contains supplementary material available at 10.1186/s12931-025-03398-0.

## Background

Chronic lung disease is a common comorbidity in people with HIV (PWH). PWH experience a greater incidence of chronic obstructive lung disease (COPD) and a faster decline in lung function than people without HIV (PWoH), even after accounting for cigarette smoking [1–3]. Emphysema is more common in PWH who smoke tobacco than in people without HIV who smoke [4]. Impairment in diffusing capacity for carbon monoxide (DLCO), with or without airflow obstruction, is the most prevalent abnormal pulmonary function finding in PWH; low DLCO may represent early emphysema, pulmonary fibrosis, or pulmonary vascular abnormalities, and is associated with a history of respiratory infections [5, 6]. Airflow obstruction and impaired diffusing capacity are both independent predictors of mortality in PWH [7].

Proposed mechanisms for the higher incidence of lung disease in PWH include direct effects of the virus, a history of pneumonia and opportunistic infections, and injury from lung toxins due to altered immune responses [8]. HIV persists in the lungs even after viral suppression and can shift macrophages to produce pro-inflammatory cytokines and proteases, disrupt cell-cell adhesion, and induce oxidative stress [9–12]. HIV-mediated lung disease is also related to dysfunctional T-cell responses as there is an association between lower CD4 + T-cell counts and COPD [13]. Immune dysregulation from HIV may enhance the toxic effects of smoking on the lungs [14]. Alveolar macrophage production of MMP9 and MMP12, immune-modulating cytokines and proteases associated with emphysema, are higher in PWH who smoke as compared to PWoH who smoke [15, 16]. It is possible that air pollution-mediated lung injury, similar to tobacco smoking injury, is amplified in the presence of HIV. In general populations, long-term exposure to fine particulate matter (PM_2.5_) is associated with accelerated decline in lung function and progression of emphysema [17, 18]. In people with COPD, exposure to higher levels of ambient air pollution is associated with increased exacerbations [19, 20]. However, there are no data regarding the effects of PM_2.5_ and ozone exposures on respiratory disease in PWH. We sought to explore associations between ambient air pollution exposures and respiratory disease in PWH and comparable populations of PWoH at increased vulnerability for HIV.

In this cross-sectional study, we focused on respiratory symptom scores as a measure of respiratory disease activity and also evaluated associations between pollution exposures and lung function. We included people with and without lung disease but hypothesized that greater 1-year average ambient air pollution exposures would be associated with worse respiratory symptoms in people with chronic lung disease and HIV.

## Methods

### Study population

We included participants of the Multicenter AIDS Cohort Study (MACS) and the Women’s Interagency HIV Study (WIHS), prospective observational cohorts of U.S. men and women, respectively, with or at increased vulnerability for HIV [21, 22], merged into the MACS/WIHS Combined Cohort Study (MWCCS) in 2020 [23]. Clinical trial number: not applicable. Individuals were eligible for this analysis if they completed pulmonary function tests (PFTs) and respiratory questionnaires at one study visit in 2017–2020 and lived in a single census tract during the year prior to testing. Individuals’ residential addresses were geocoded and assigned to census tracts using ArcGIS (Esri, Redlands, CA), which were then linked to census tract-level daily average ambient fine particulate matter (PM_2.5_) and 8-hour maximum ozone (O_3_) concentrations from the U.S. Environmental Protection Agency’s Community Multiscale Air Quality Modeling System (CMAQ) [24]. To determine how long an individual had lived at a given location, due to MACS and WIHS protocol differences, we used self-reported duration of residence for men and longitudinal (annual) geocoding data for women.

### Exposures and outcomes

Concentrations of PM_2.5_ (µg/m³) and O_3_ (parts per billion, ppb) were the two exposures of interest, each averaged across the year preceding the date of the PFTs. Since we had one year of confirmed location data, we selected one-year average pollution estimates to incorporate potential lag or cumulative effects over the given exposure time interval. A total of six respiratory-related outcomes were examined. Four were measures of lung function: postbronchodilator % predicted forced expiratory volume at 1 s (FEV_1_), % predicted forced vital capacity (FVC), FEV_1_/FVC ratio, and % predicted DLCO. Two were measures of symptom burden: St. George’s Respiratory Questionnaire (SGRQ) score [25] and modified Medical Research Council (mMRC) dyspnea scale score [26]. The SGRQ total score was used (0–100 range), with higher scores indicating worse respiratory health status [27]. Ordinal mMRC scores were whole numbers from 0 (“I only get breathless with strenuous exercise”) to 4 (“I am too breathless to leave the house, or I am breathless when dressing”) [28]. We also created a binary mMRC variable reflecting a low burden of symptoms (score 0 or 1) or a high burden of symptoms (score 2, 3, or 4), as higher scores predict mortality independent of lung function [29]. Spirometry measurements were made with an EasyOne Pro or Easy-on-PC spirometer (ndd Medizintechnik AG, Zurich, Switzerland); bronchodilation was by albuterol (360 µg via metered-dose inhaler). Quality assessment was according to American Thoracic Society/European Respiratory Society (ATS/ERS) standards [30], and equations derived from the Third National Health and Nutrition Examination Survey (NHANES) were used to calculate spirometry reference values [31]. DLCO measurements were made with an EasyOne Pro device and adjusted for hemoglobin and carboxyhemoglobin following the ATS/ERS standards; [32] equations from NHANES I were used to calculate DLCO reference values [32, 33]. Spirometry and DLCO reference equations were chosen to align with previously published lung function analyses in these cohorts [34, 35]. 

### Statistical analysis

Linear regression models were used to quantify unadjusted and adjusted associations between selected increases in the two exposures (3 ppb for O_3_, and 1 µg/m³ for PM_2.5_) and the six outcomes, separately for MACS PWH, MACS PWoH, WIHS PWH, and WIHS PWoH. We also performed sensitivity analyses that pooled PWH and PWoH in each cohort for each outcome and analyzed an interaction between HIV serostatus and PM_2.5_ or O_3_ exposure. Covariates posited a priori as potentially associated with the outcomes were included in adjusted models: age (5-knot restricted cubic spline), race (Black, White, or other), Hispanic ethnicity (binary), current cannabis use (binary), current tobacco smoking (binary), smoking pack-years history (continuous), and body mass index (continuous). In models restricted to PWH, CD4 count (continuous, cells/mm^3^), CD4/CD8 ratio (continuous), and log-transformed HIV viral load (copies/mL), obtained within six months of the PFT study visit, were additionally included. For the binary mMRC outcome, we used logistic regression, with all other modeling details identical. Because we hypothesized that people with underlying chronic lung disease would be most susceptible to the effects of ambient pollution exposure, we next conducted two subgroup analyses, the first restricted to individuals with COPD (defined as post-bronchodilator FEV_1_/FVC ratio < 0.7) [36], and the other restricted to individuals with impaired diffusing capacity (DLCO < 80% predicted, as previously defined in this population) [34]. The subgroup analyses were not stratified by cohort (MACS, WIHS) due to smaller sample sizes; as such, sex (cohort) was included in regression models which were otherwise as specified above, including separate models for MACS/WIHS PWH and MACS/WIHS PWoH. Missing covariate data were minimal; a small number of values were carried forward from the prior study visit. Analyses were completed in SAS version 9.4 (SAS Institute Inc., Cary, NC).

## Results

### Baseline characteristics

In the MACS cohort, 338 men met inclusion criteria [Table 1]. One hundred forty-six (43%) were PWH, with well-controlled HIV (median viral load undetectable and median CD4 + T-cell count 674 cells/mm^3^). In the MACS, PWH were younger (59 vs. 64 years), were more likely to be people who currently smoke (15% vs. 10%), had greater smoking history (median of 16 pack-years vs. 9 pack-years), and were more likely to have COPD (12% vs. 6%), as compared to PWoH. In MACS there was a higher percentage of Blacks and Hispanics in the PWH group compared to PWoH (30% vs. 12% and 16% vs. 4%, respectively). In the WIHS cohort, 1073 women met inclusion criteria. PWH in the WIHS also had well-controlled HIV, with median viral load undetectable and CD4 + T-cell count 726 cells/mm^3^. PWH and PWoH in WIHS were of similar age (52 vs. 50 years, respectively) and had similar smoking history, current smoking status, and prevalence of COPD.

**Table 1:** Characteristics of included MACS and WIHS participants, by HIV serostatus

Cohort characteristics	MACS			WIHS		
	Total	PWH	PWoH	Total	PWH	PWoH
No. of participants	338	146	192	1073	769	304
Age in years, median (IQR)	62 (56–68)	59 (53–65)	64 (58.5–70)	52 (44–58)	52 (45–58)	50 (42–57)
Sex, No. (%)						
Female				1073 (100.0)	769 (100.0)	304 (100.0)
Male	338 (100.0)	146 (100.0)	192 (100.0)			
Race, No. (%)						
Black	66 (19.5)	43 (29.5)	23 (12.0)	707 (65.9)	502 (65.3)	205 (67.4)
White	242 (71.6)	81 (55.5)	161 (83.9)	107 (10.0)	87 (11.3)	20 (6.6)
Other	30 (8.9)	22 (15.1)	8 (4.2)	259 (24.1)	180 (23.4)	79 (26.0)
Hispanic ethnicity, No. (%)	31 (9.2)	24 (16.4)	7 (3.6)	138 (12.9)	96 (12.5)	42 (13.8)
<=High school education, No. (%)	36 (10.7)	22 (15.1)	14 (7.3)	669 (62.4)	494 (64.2)	175 (57.8)
Smoking status, No. (%)						
Never	128 (38.4)	56 (39.2)	72 (37.9)	372 (34.7)	272 (35.4)	100 (32.9)
Current	39 (11.7)	21 (14.7)	18 (9.5)	382 (35.6)	266 (34.6)	116 (38.2)
Former	166 (49.8)	66 (46.2)	100 (52.6)	319 (29.7)	231 (30.0)	88 (28.9)
Pack-years of smoking (median, IQR)*	11.9 (0.4–31.1)	15.6 (4.4–28.3)	9.1 (0.1–31.5)	9.9 (3.9–17.1)	9.7 (4.0–16.8)	10.3 (3.9–17.8)
BMI (median, IQR)	26.9 (24.6–29.8)	26.5 (23.9–29.7)	27.2 (25.2–29.8)	31.9 (26.7–38.5)	31.6 (24.6–38.0)	32.4 (27.1–39.0)
HIV markers, median (IQR)						
HIV viral load (copies/mL)		ND (ND–20)			ND (ND–20)	
CD4 count (cells/mm^3^)		674 (494–878)			726 (514–955)	
CD4/CD8 ratio		0.9 (0.6–1.3)			1.0 (0.6–1.4)	
FVC % predicted, mean (SD)	94 (15)	96 (14)	93 (15)	89 (18)	89 (17)	91 (20)
FEV_1_ % predicted, mean (SD)	97 (16)	98 (16)	97 (17)	89 (19)	89 (18)	91 (20)
FEV_1_/FVC, mean (SD)	0.78 (0.07)	0.78 (0.07)	0.78 (0.06)	0.80 (0.09)	0.80 (0.08)	0.80 (0.09)
DLCO % predicted, mean (SD)	85 (14)	86 (15)	85 (13)	86 (16)	84 (16)	92 (17)
COPD, No. (%)	29 (8.6)	17 (11.6)	12 (6.3)	111 (10.3)	79 (10.3)	32 (10.5)
Air pollution						
O_3_, ppb, median (IQR)	39.0 (36.9–39.9)	38.7 (36.7–39.9)	39.3 (37.0–40.0)	36.4 (34.1–37.8)	36.4 (34.1–37.8)	36.2 (34.0–37.8)
O_3_, ppb, mean (SD)	39.2 (3.2)	39.1 (3.3)	39.3 (3.2)	35.8 (3.1)	35.9 (3.0)	35.5 (3.3)
PM_2.5_ µg/m^3^, median (IQR)	9.4 (8.5–10.3)	9.4 (8.6–10.4)	9.3 (8.4–10.3)	9.2 (8.7–10.1)	9.2 (8.7–10.1)	9.3 (8.7–10.0)
PM_2.5_ µg/m^3^, mean (SD)	9.5 (1.3)	9.5 (1.2)	9.5 (1.3)	9.4 (1.2)	9.4 (1.2)	9.4 (1.1)

*MACS* Multicenter AIDS Cohort Study, *WIHS* Women’s Interagency HIV Study, *PWH* People with HIV, *PWoH* People without HIV, *IQR* Interquartile range, *SD* Standard deviation, *BMI* Body mass index, *ND* Not detected, *FVC* Forced vital capacity, *FEV*_1_ Forced expiratory volume at 1 second, *DLCO* Diffusion capacity for carbon monoxide, *COPD* Chronic obstructive pulmonary disease, *PM*_2.5_ Particulate matter <2.5 microns, *O*_3_ Ozone, *ppb* Parts per billion

*n*=5 missing smoking status in MACS

*n*=25 and 672 missing DLCO % predicted in MACS and WIHS, respectively

*Pack-years of smoking among current and former smokers

As compared to MACS participants, WIHS participants were younger (52 years vs. 62 years), were more often Black (66% vs. 20%), were more likely to have no more than a high school education (62% vs. 11%), had higher BMI, and were more likely to be people who currently smoke (36% vs. 12%). The annual average pollution exposures between MACS and WIHS participants were similar, with median O_3_ exposures 39.0 and 36.4 ppb, and median PM_2.5_ exposures 9.4 and 9.2 µg/m^3^, respectively. Median pollution exposures were also similar between PWH and PWoH in both cohorts.

Pollution levels by site are listed in Supplementary Table 1. There were generally higher levels of pollution exposures at the California sites whereas the other sites showed similar distributions.

### Pollution exposure and lung function

We assessed associations between lung function and 1-year average PM_2.5_ and O_3_ exposures [Table 2]. While some findings reached statistical significance, there were no clear trends. In the MACS, PWoH had slightly higher % predicted DLCO with higher O_3_ exposure (2.39%, 95% confidence interval [CI] 0.65 to 4.14), while in the WIHS, PWH had higher % predicted DLCO with higher O_3_ exposure (1.36%, 96% CI 0.2 to 2.51). In the WIHS, higher PM_2.5_ exposure was associated with lower FEV_1_/FVC ratio in PWoH (−0.012, 95% CI −0.021 to −0.003) but not in PWH. In summary, we did not find any clinically significant associations between 1-year average PM_2.5_ or O_3_ exposures and cross-sectional lung function.

**Table 2:** Relationships between PM_2.5_ and O_3_ exposures and lung function, by cohort and HIV serostatus

		MACS N=338 (PWoH 192, PWH 146) Effect estimate^a^ (95% CI)		WIHS N=1073 (PWoH 304, PWH 769) Effect estimate^a^ (95% CI)	
		Unadjusted	Adjusted^b^	Unadjusted	Adjusted^b^
PM_2.5_ 1 µg/m^3^ increase	FEV_1_/FVC				
	*PWoH*	0.001 (−0.006, 0.008)	−0.001 (−0.008, 0.006)	**−0.012 (−0.021, −0.002)**	**−0.012 (−0.021, −0.003)**
	*PWH*	**0.009 (0, 0.019)**	0.003 (−0.006, 0.012)	−0.002 (−0.007, 0.004)	0.001 (−0.004, 0.006)
	% predicted FEV_1_				
	*PWoH*	0.40 (−1.41, 2.22)	0.71 (−1.13, 2.55)	−0.04 (−2.07, 1.98)	−0.21 (−2.18, 1.76)
	*PWH*	1.38 (−0.72, 3.49)	1.83 (−0.43, 4.08)	0.45 (−0.66, 1.56)	0.65 (−0.46, 1.76)
	% predicted FVC				
	*PWoH*	0.98 (−0.62, 2.58)	0.93 (−0.70, 2.55)	1.47 (−0.57, 3.50)	1.31 (−0.70, 3.33)
	*PWH*	0.77 (−1.13, 2.68)	1.39 (−0.55, 3.32)	0.46 (−0.55, 1.48)	0.46 (−0.56, 1.47)
	% predicted DLCO				
	*PWoH*	1.38 (−0.07, 2.83)	0.73 (−0.74, 2.19)	0.36 (−1.96, 2.68)	−0.93 (−3.27, 1.41)
	*PWH*	1.27 (−0.85, 3.39)	1.60 (−0.57, 3.77)	0.38 (−0.83, 1.59)	**1.36 (0.20, 2.51) **
O_3_ 3 ppb increase	FEV_1_/FVC				
	*PWoH*	0.004 (−0.005 0.012)	0.001 (−0.007, 0.01)	−0.002 (−0.011, 0.008)	−0.003 (−0.012, 0.006)
	*PWH*	**0.013 (0.002, 0.023) **	0.009 (−0.0001, 0.018)	−0.0002 (−0.006, 0.006)	−0.004 (−0.01, 0.002)
	% predicted FEV_1_				
	*PWoH*	0.03 (−2.19, 2.24)	0.82 (−1.44, 3.08)	−1.17 (−3.20, 0.87)	−0.88 (−2.85, 1.09)
	*PWH*	1.01 (−1.34, 3.37)	1.51 (−0.83, 3.86)	−0.15 (−1.47, 1.17)	−0.13 (−1.45, 1.20)
	% predicted FVC				
	*PWoH*	−0.15 (−2.11, 1.81)	0.67 (−1.34, 2.67)	−0.69 (−2.74, 1.37)	−0.51 (−2.53, 1.51)
	*PWH*	−0.29 (−2.42, 1.84)	0.35 (−1.66, 2.37)	0.25 (−0.96, 1.45)	0.27 (−0.93, 1.48)
	% predicted DLCO				
	*PWoH*	**2.28 (0.54, 4.02) **	**2.39 (0.63, 4.14) **	−0.03 (−2.24, 2.18)	−0.15 (−2.31, 2.00)
	*PWH*	0.89 (−1.52, 3.30)	1.76 (−0.55, 4.07)	0.33 (−1.03, 1.69)	−0.80 (−2.10, 0.50)

*MACS* Multicenter AIDS Cohort Study, *WIHS* Women’s Interagency HIV Study, *PWH* people with HIV, *PWoH* People without HIV, *CI* Confidence interval, *FVC* Forced vital capacity, *FEV*_1_Forced expiratory volume at 1 second, *DLCO* Diffusion capacity for carbon monoxide, *PM*_2.5_ Particulate matter <2.5 microns, *O*_3_ Ozone, *ppb* Parts per billion; boldface entries indicate a statistically significant correlation

^a^ Difference in lung function for 1 µg/m^3^ increase in PM_2.5_exposure or 3 ppb increase in O_3_ exposure

^b^ Separate models for MACS PWH, MACS PWoH, WIHS PWH, and WIHS PWoH adjusted for age, race, ethnicity, current smoking, smoking pack-years, and BMI; PWH models also adjusted for CD4 count, CD4/CD8 ratio, HIV viral load

### Pollution exposure and symptom scores

In both MACS and WIHS populations, there were no notable trends in respiratory symptom scores with higher PM_2.5_ or O_3_ exposure when stratified by HIV serostatus [Table 3]. Both PWH and PWoH in the WIHS had worse SGRQ scores with higher PM_2.5_ exposure; however, these increases did not remain significant after adjustment for covariates. PWoH in the WIHS had worse mMRC scores with greater PM_2.5_ exposure when mMRC was specified as a binary outcome. SGRQ scores were slightly lower with greater O_3_ exposure only in PWoH in the WIHS. Among MACS participants, there were no significant associations between pollution exposure and respiratory symptom scores.

**Table 3:** Relationships between PM_2.5_ and O_3_ exposures and respiratory symptom scores, by cohort and HIV serostatus

		MACS N=338 (PWoH 192, PWH 146) Effect estimate^a^ (95% CI)		WIHS N=1073 (PWoH 304, PWH 769) Effect estimate^a^ (95% CI)	
		**Unadjusted**	**Adjusted** ^b^	**Unadjusted**	**Adjusted** ^b^
PM_2.5_ 1 µg/m^3^ increase	SGRQ				
	*PWoH*	−0.18 (−1.30, 0.95)	0.22 (−0.88, 1.32)	**1.63 (0.13, 3.14)**	1.37 (−0.05, 2.79)
	*PWH*	0.69 (−1.04, 2.43)	−0.04 (−1.92, 1.85)	**1.24 (0.24, 2.24)**	0.61 (−0.37, 1.59)
	mMRC				
	*PWoH*	0.01 (−0.07, 0.09)	−0.02 (−0.09, 0.05)	0.07 (−0.06, 0.19)	0.05 (−0.07, 0.17)
	*PWH*	0.08 (−0.04, 0.20)	−0.03 (−0.15, 0.09)	0.07 (−0.01, 0.15)	0.05 (−0.03, 0.12)
	mMRC binary^c^				
	*PWoH*	0.98 (0.61, 1.58)	0.64 (0.25, 1.66)	1.24 (0.99, 1.55)	**1.36 (1.04, 1.78)**
	*PWH*	1.37 (0.89, 2.11)	0.95 (0.51, 1.80)	1.09 (0.97, 1.24)	1.06 (0.92, 1.21)
O_3_ 3 ppb increase	SGRQ				
	*PWoH*	−0.51 (−1.91, 0.90)	−0.002 (−1.40, 1.39)	**−1.78 (−3.30, −0.27)**	**−1.72 (−3.13, −0.31)**
	*PWH*	0.15 (−1.81, 2.10)	−0.54 (−2.50, 1.41)	−0.31 (−1.49, 0.89)	0.11 (−1.06, 1.28)
	mMRC				
	*PWoH*	0.02 (−0.08, 0.11)	−0.01 (−0.09, 0.08)	−0.02 (−0.14, 0.11)	−0.03 (−0.15, 0.09)
	*PWH*	0.08 (−0.05, 0.21)	0.03 (−0.10, 0.17)	0.04 (−0.06, 0.13)	0.05 (−0.04, 0.15)
	mMRC binary^c^				
	*PWoH*	1.07 (0.62, 1.86)	0.45 (0.12, 1.77)	0.92 (0.73, 1.15)	0.85 (0.65, 1.10)
	*PWH*	1.42 (0.94, 2.14)	1.48 (0.75, 2.91)	1.05 (0.91, 1.22)	1.09 (0.93, 1.28)

*MACS* Multicenter AIDS Cohort Study, *WIHS* Women’s Interagency HIV Study, *PWH* people with HIV, *PWoH* people without HIV, *CI* Confidence interval, *SGRQ* St. George’s Respiratory Questionnaire score, *mMRC* Modified Medical Research Council dyspnea scale score, *PM*_2.5_ Particulate matter <2.5 microns, *O*_3_ Ozone, *ppb* Parts per billion; boldface entries indicate a statistically significant correlation

^a^ Difference in SGRQ and mMRC for 1 µg/m^3^ increase in PM_2.5_exposure or 3 ppb increase in O_3_ exposure

^b^ Separate models for MACS PWH, MACS PWoH, WIHS PWH, and WIHS adjusted for age, race, ethnicity, current smoking, smoking pack-years, and BMI; PWH models also adjusted for CD4 count, CD4/CD8 ratio, HIV viral load

^c^ Binary mMRC outcome: high (2–4) vs. low (0–1), estimate is odds ratio from logistic regression

### Symptoms in participants with COPD

Within the MACS and WIHS cohorts, there were a total of 140 participants diagnosed with COPD, 96 PWH and 44 PWoH [Table 4]. In the COPD group 79% were women, reflective of the larger number of WIHS than MACS participants included in the overall population. More PWH had no smoking history compared to PWoH (26% vs. 18%) and smoking history was less in PWH than in PWoH (median of 14 vs. 22 pack-years). Within the COPD subgroup, a 1 µg/m^3^ increase in 1-year average exposure to PM_2.5_ was associated with an increase in 4.11 points in the SGRQ score in PWH (95% CI 0.57 to 7.65) [Table 5]. Worse SGRQ score with greater PM_2.5_ exposure persisted in the PWH group after adjusting for relevant covariates (4.04 points, 95% CI 0.36 to 7.72). The association between poor respiratory symptom scores and PM_2.5_ exposure in PWH persisted after additionally adjusting for degree of airflow obstruction (FEV_1_% predicted) (3.62 points, 95% CI 0.07 to 7.17). In contrast, there were no significant associations between PM_2.5_ exposure and SGRQ score in the PWoH group.

**Table 4:** Characteristics of included participants in COPD and impaired DLCO subgroups, by HIV serostatus

Cohort characteristics	COPD			Impaired Diffusion Capacity (DLCO <80% predicted)		
	Total	PWH	PWoH	Total	PWH	PWoH
No. of participants	140	96	44	240	149	91
Age, median (IQR), y	56 (49–63)	55 (47–61)	61 (53–65)	59 (53–65)	57 (50–61)	64 (56–71)
Sex, No. (%)						
Female	111 (79.3)	79 (82.3)	32 (72.7)	135 (56.3)	110 (73.8)	25 (27.5)
Male	29 (20.7)	17 (17.7)	12 (27.3)	105 (43.7)	39 (26.2)	66 (72.5)
Race, No. (%)						
Black	77 (55.0)	53 (55.2)	24 (54.5)	108 (45.0)	83 (55.7)	25 (27.5)
White	34 (24.3)	22 (22.9)	12 (27.3)	97 (40.4)	40 (26.8)	57 (62.6)
Other	29 (20.7)	21 (21.9)	8 (18.2)	35 (14.6)	26 (17.4)	9 (9.9)
Hispanic ethnicity, No. (%)	10 (7.1)	7 (7.3)	3 (6.8)	26 (10.8)	22 (14.8)	4 (4.4)
<=High school education, No. (%)	70 (50.0)	52 (54.2)	18 (40.9)	107 (44.6)	82 (55.0)	25 (27.5)
Smoking status, No. (%)						
Never	32 (23.2)	24 (25.5)	8 (18.2)	71 (29.7)	46 (31.1)	25 (27.5)
Current	59 (42.3)	42 (44.7)	17 (38.6)	81 (33.9)	62 (41.9)	19 (20.9)
Former	47 (34.1)	28 (29.8)	19 (43.2)	87 (36.4)	40 (27.0)	47 (51.6)
Pack-years of smoking (median, IQR)*	15.5 (5.7–30.2)	14.3 (6.1–26.9)	21.9 (4.8–40.3)	13.3 (4.4–26.5)	12.2 (5.0–20.7)	22.0 (1.1–38.3)
BMI (median, IQR)	29.4 (24.2–34.0)	28.4 (23.9–32.6)	31.2 (25.4–36.7)	28.3 (25.2–33.7)	28.1 (24.4–33.8)	28.6 (25.7–33.1)
HIV markers, median (IQR)						
HIV viral load (copies/mL)		ND (ND–20)			ND (ND–20)	
CD4 count (cells/mm^3^)		704 (480–876)			652 (459–874)	
CD4/CD8 ratio		1.0 (0.7–1.3)			0.7 (0.5–1.2)	
FVC % predicted, mean (SD)	91 (25)	91 (21)	91 (32)	86 (15)	85 (15)	87 (16)
FEV_1_ % predicted, mean (SD)	71 (17)	71 (16)	70 (20)	87 (17)	86 (16)	88 (17)
FEV_1_/FVC, mean (SD)	0.62 (0.08)	0.62 (0.08)	0.60 (0.09)	0.79 (0.07)	0.80 (0.07)	0.77 (0.08)
DLCO % predicted, mean (SD)	80 (18)	79 (20)	82 (15)	69 (8)	69 (8)	71 (7)
COPD, No. (%)				25 (10.4)	14 (9.4)	11 (12.1)
Air pollution						
O_3_, ppb, median (IQR)	36.9 (34.9–38.1)	36.7 (34.8–37.9)	37.1 (35.1–38.4)	37.9 (35.9–39.3)	37.7 (35.6–39.1)	38.4 (36.5–39.5)
O_3_, ppb, mean (SD)	36.4 (3.1)	36.3 (3.0)	36.7 (3.5)	37.2 (3.5)	36.8 (3.5)	38.0 (3.5)
PM_2.5_ µg/m^3^, median (IQR)	9.5 (8.8–10.2)	9.4 (8.7–10.1)	9.9 (8.9–10.4)	9.3 (8.6–10.3)	9.5 (8.6–10.3)	9.3 (8.4–10.3)
PM_2.5_ µg/m^3^, mean (SD)	9.6 (1.2)	9.4 (1.1)	9.8 (1.5)	9.6 (1.4)	9.6 (1.4)	9.5 (1.3)

*MACS* Multicenter AIDS Cohort Study, *WIHS* Women’s Interagency HIV Study, *PWH* People with HIV, *PWoH* People without HIV, *IQR* Interquartile range, *SD* Standard deviation, *BMI* Body mass index, *ND* Not detected, *FVC* Forced vital capacity, *FEV*_1_ Forced expiratory volume at 1 second, *DLCO* Diffusion capacity for carbon monoxide, *COPD* Chronic obstructive pulmonary disease, *PM*_2.5_ Particulate matter <2.5 microns, *O*_3_ Ozone, *ppb* Parts per billion

*n*=2, 1 missing smoking status in COPD and DLCO <80% predicted, respectively

*n*=87 missing DLCO % predicted in COPD

*Pack-years of smoking among current and former smokers

**Table 5:** Relationships between PM_2.5_ and O_3_ exposures and respiratory symptom scores in participants with COPD, by HIV serostatus

		MACS/WIHS COPD Subgroup N=140 (PWoH 96, PWH 44) Effect estimate^a^ (95% CI)		
		Unadjusted	Adjusted^b^	Adjusted for FEV_1_ % predicted^c^
PM_2.5_ 1 µg/m^3^ increase	SGRQ			
	*PWoH*	2.58 (−0.92, 6.07)	1.80 (−1.55, 5.16)	1.99 (−1.36, 5.34)
	*PWH*	**4.11 (0.57, 7.65)**	**4.04 (0.36, 7.72)**	**3.62 (0.07, 7.17)**
	mMRC			
	*PWoH*	0.08 (−0.1, 0.37)	−0.11 (−0.34, 0.13)	−0.12 (−0.35, 0.11)
	*PWH*	**0.26 (0.01, 0.51)**	**0.28 (0.02, 0.53)**	**0.27 (0.01, 0.52)**
	mMRC binary^d^			
	*PWoH*	1.30 (0.84, 2.02)	3.96 (0.52, 30.05)	
	*PWH*	**1.55 (1.02, 2.36)**	1.57 (0.96, 2.57)	
O_3_ 3 ppb increase	SGRQ			
	*PWoH*	**−4.70 (−8.98, −0.42)**	−3.00 (−7.70, 1.70)	−3.18 (−7.86, 1.50)
	*PWH*	−1.06 (−4.95, 2.83)	−1.33 (−5.46, 2.80)	−1.76 (−5.72, 2.21)
	mMRC			
	*PWoH*	0.02 (−0.35, 0.38)	−0.01 (−0.34, 0.32)	0.005 (−0.32, 0.33)
	*PWH*	−0.13 (−0.41, 0.14)	−0.09 (−0.38, 0.20)	−0.10 (−0.39, 0.19)
	mMRC binary^d^			
	*PWoH*	1.01 (0.60, 1.69)	0.95 (0.17, 5.42)	
	*PWH*	0.71 (0.45, 1.11)	0.72 (0.40, 1.30)	

*MACS* Multicenter AIDS Cohort Study, *WIHS* Women’s Interagency HIV Study, *PWH* People with HIV, *PWoH* People without HIV, *CI* Confidence interval, *SGRQ* St. George’s Respiratory Questionnaire score, *mMRC* Modified Medical Research Council dyspnea scale score, *FEV*_1_ Forced expiratory volume at 1 second, *PM*_2.5_ Particulate matter <2.5 microns, *O*_3_ Ozone, *ppb* Parts per billion; boldface entries indicate a statistically significant correlation

^a^ Difference in SGRQ and mMRC for 1 µg/m^3^ increase in PM_2.5_exposure or 3 ppb increase in O_3_ exposure

^b^ Separate models for MACS/WIHS PWH and MACS/WIHS PWoH adjusted for sex, age, race, ethnicity, current smoking, smoking pack-years, BMI; PWH model also adjusted for CD4 count, CD4/CD8 ratio, HIV viral load

^c^ Adjusted for above variables plus FEV_1_ % predicted

^d^ Binary mMRC outcome: high (2–4) vs. low (0–1), estimate is odds ratio from logistic regression

Similar results were found with mMRC scores: for a 1 µg/m^3^ increase in 1-year average PM_2.5_ exposure, mMRC scores worsened by 0.26 points in PWH with COPD (95% CI 0.01–0.51); the association persisted after adjustment for covariates including baseline airflow obstruction (0.27 points, 95% CI 0.01 to 0.52). Higher PM_2.5_ exposure was also associated with higher mMRC (binary specification), although this association did not hold after adjustment for covariates. There was no significant association between PM_2.5_ exposure and mMRC score in PWoH with COPD.

Unlike PM_2.5_, there were no significant associations between higher O_3_ exposure and respiratory symptoms in either PWH with COPD or PWoH with COPD, aside from lower SGRQ in PWoH with increased O_3_ exposure on unadjusted analyses only.

#### Symptoms in participants with impaired DLCO

There were a total of 240 participants in MACS and WIHS with DLCO < 80% predicted, 149 PWH and 91 PWoH [Table 4]. Within this subgroup, 10% also had COPD by spirometry. In the impaired DLCO group, smoking history was less in PWH than in PWoH (median 12 pack-years vs. 22 pack-years).

In PWH with impaired DLCO, there was an association between 1 µg/m^3^ greater 1-year average PM_2.5_ exposure and worse respiratory symptoms, both by SGRQ (2.77 points, 95% CI 0.81 to 4.73) and mMRC (0.21 points, 95% CI 0.05 to 0.36) [Table 6]. This finding persisted after adjustment for covariates (SGRQ 2.14 points, 95% CI 0.2 to 4.08; and mMRC 0.16 points, 95% CI 0.02 to 0.29). Similarly, increased PM_2.5_ exposure was associated with higher mMRC (binary specification), although the estimate for PWH was attenuated after adjustment for HIV variables. In PWoH with impaired DLCO, there was no significant association between increased PM_2.5_ exposure and respiratory symptom scores. With higher O_3_ exposure, there were no significant differences in respiratory symptoms with increased O_3_ exposure in either PWH or PWoH in the adjusted models.

**Table 6:** Relationships between PM_2.5_ and O_3_ exposures and respiratory symptom scores in impaired diffusion capacity participants, by HIV serostatus

		MACS/WIHS Impaired Diffusion Capacity Subgroup *N*=240 (PWoH 91, PWH 149) Effect estimate^a^ (95% CI)	
		Unadjusted	Adjusted^b^
PM_2.5_ 1 µg/m^3^ increase	SGRQ		
	*PWoH*	0.17 (−2.33, 2.66)	−0.04 (−2.40, 2.33)
	*PWH*	**2.77 (0.81, 4.73)**	**2.14 (0.20, 4.08)**
	mMRC		
	*PWoH*	0.06 (−0.12, 0.25)	0.01 (−0.14, 0.17)
	*PWH*	**0.21 (0.05, 0.36)**	**0.16 (0.02, 0.29)**
	mMRC binary^c^		
	*PWoH*	1.18 (0.82, 1.70)	1.11 (0.54, 2.28)
	*PWH*	**1.37 (1.06, 1.78)**	1.37 (0.99, 1.87)
O_3_ 3 ppb increase	SGRQ		
	*PWoH*	**−4.55 (−7.27, −1.83)**	−2.58 (−5.49, 0.34)
	*PWH*	−1.33 (−3.75, 1.10)	−0.32 (−3.04, 2.41)
	mMRC		
	*PWoH*	**−0.27 (−0.47, −0.07)**	−0.09 (−0.28, 0.10)
	*PWH*	−0.09 (−0.28, 0.10)	−0.02 (−0.21, 0.17)
	mMRC binary^c^		
	*PWoH*	**0.54 (0.33, 0.88)**	0.57 (0.22, 1.48)
	*PWH*	0.89 (0.67, 1.18)	0.96 (0.64, 1.44)

*MACS* Multicenter AIDS Cohort Study, *WIHS* Women’s Interagency HIV Study, *PWH* People with HIV, *PWoH* People without HIV, *CI* Confidence interval, *SGRQ* St. George’s Respiratory Questionnaire score, *mMRC* Modified Medical Research Council dyspnea scale score, *PM*_2.5_ Particulate matter <2.5 microns, *O*_3_ Ozone, *ppb* Parts per billion; boldface entries indicate a statistically significant correlation

^a^ Difference in SGRQ and mMRC for 1 µg/m^3^ increase in PM_2.5_exposure or 3 ppb increase in O_3_ exposure

^b^ Separate models for MACS/WIHS PWH and MACS/WIHS PWoH adjusted for sex, age, race, ethnicity, current smoking, smoking pack-years, BMI; PWH model also adjusted for CD4 count, CD4/CD8 ratio, HIV viral load

^c^ Binary mMRC outcome: high (2–4) vs. low (0–1), estimate is odds ratio from logistic regression

### Sensitivity analyses pooling PWH and PWoH

In sensitivity analyses pooling PWH and PWoH groups, the p-value for the interaction term between HIV serostatus and the pollution exposure reached statistical significance (< 0.05) only for PM_2.5_ exposure and lower FEV1/FVC ratio. The p-value was not statistically significant for the interaction term between HIV serostatus and PM_2.5_ or O_3_ exposure in any other lung function measure or symptom score outcome in the MACS or WIHS, or in any symptom score outcome in the COPD and impaired diffusion subgroups. These data are not adjusted for HIV-related covariates (Supplementary Tables 2–4).

## Discussion

We found that among participants in the MACS and WIHS cohorts, greater exposure to PM_2.5_ was associated with worse respiratory symptoms in PWH with COPD or impaired diffusion capacity, while this was not the case in PWoH with similar lung disease characteristics. To our knowledge, our study is the first to examine the potential negative effects of the combination of HIV infection and PM_2.5_ air pollution, using standardized questionnaires to measure respiratory symptoms, impact, and functional disability, and finding an enhanced negative effect in those with underlying COPD or impaired DLCO. These results demonstrate the relevance of air pollution exposure among susceptible populations, as the average PM_2.5_ concentrations were not excessively high. It is notable that an increase of 1 µg/m^3^ in average outdoor pollution exposure was associated with a 4-point increase in SGRQ, the threshold considered clinically relevant for patient outcomes and management [27]. Worse SGRQ scores are associated with increased healthcare utilization, hospital readmissions, and exacerbations [37, 38]. Our data supports the American Thoracic Society findings from the Health of the Air Report [39] that calls for a reduction in outdoor air standards to 8 µg/m^3^, and provides evidence that lower standards may result in a reduction in respiratory morbidities.

Our results align with prior small studies showing that PWH may experience worse respiratory symptoms when exposed to other forms of air pollution. In a study in Malawi [40], respiratory symptoms such as breathlessness and cough were common in PWH who were exposed to household air pollution (HAP). In PWH exposed to HAP, there was a trend toward increased proinflammatory cytokines and vascular injury markers, as well as lower levels of IL-2 and IL-16, cytokines associated with HIV clearance. Inflammation, vascular injury, and viral persistence are potential mechanisms of HAP-related COPD in PWH. Similarly, a study assessing carbon monoxide (CO) exposure from biomass fuel use in Uganda demonstrated that higher CO exposure was associated with worse respiratory symptoms in PWH but not in PWoH [41]. 

Interestingly, we found that while PM_2.5_ exposure was associated with worse symptoms in PWH with lung disease, O_3_ exposure was not. Although this is in keeping with a study showing that short-term exposure to PM_2.5_, but not O_3_ or other pollutants, was associated with COPD exacerbation [42], our results are in contrast to a SubPopulations and InteRmediate Outcome Measures in COPD (SPIROMICS) study wherein greater 10-year O_3_ exposure was associated with COPD morbidity, including reduced lung function, worse respiratory symptoms, increased exacerbations, and greater emphysema [14]. However, in the SPIROMICS study, when PM_2.5_ exposure was included in the model, there was no longer a significant association between O_3_ exposure and SGRQ score or exacerbations. It is possible that particulate exposure is more closely associated with airways disease and respiratory symptoms, while O_3_ contributes to progression of emphysema [18, 43, 44]. 

We did not find that greater pollution exposure was associated with worse respiratory symptoms in the overall population or in PWoH with chronic lung disease. While it is generally accepted that air pollution worsens outcomes in COPD, the data are variable based on type and degree of exposure: for example, a meta-analysis of studies of air pollution in COPD showed significant heterogeneity in the effects of PM_2.5_ on respiratory symptoms, and no overall association between gaseous pollutants including O_3_ and either lung function or symptoms [45]. Our negative results in the overall population and the PWoH with chronic lung disease subgroup may be related to narrow IQR ranges of pollution exposures, 1-year duration of exposure estimation, and limited sample size. Although our study was underpowered to detect small differences, we found the strongest associations in people with multiple susceptibilities to air pollution (HIV plus chronic lung disease). We speculate that the effects of air pollution are amplified in the setting of underlying airway inflammation, as mediated by both COPD and HIV.

We did not find any clinically significant associations between 1-year average PM_2.5_ or O_3_ exposures and cross-sectional lung function. Our findings are in concordance with some prior cohort studies of air pollution and lung function that showed inconclusive results [46]. We surmise that our results are attributable to the multitude of factors beyond air pollution exposure that contribute to acquired adult lung function and that could not be accounted for in this study, such as childhood respiratory disease, nutritional factors, and occupational exposures. Future analyses of longitudinal lung function in the MACS and WIHS cohorts will be informative to determine if ambient pollution exposure is associated with loss of lung function in these populations, and if HIV serostatus or the presence of baseline chronic lung disease modify any associations.

This cross-sectional study has several limitations which impact potential conclusions about air pollution and lung function. We acknowledge pollution exposure estimates using residential address may be imprecise; however, previous studies comparing pollution estimates using residential address versus time-activity patterns have shown high correlations both in degree of exposure and associated health effects [47]. In addition, there is precedent for using residential address as well as 1-year pollution exposure estimates in the COPD and air pollution literature [48]. 

Another limitation of our study is the use of two cohorts with inherent differences between the populations, most notably sex but also COPD prevalence. As with other studies utilizing these cohorts [49, 50], we chose to stratify by sex (cohort) and, in the subgroup analyses with small sample sizes, combine the MACS and WIHS participants while adjusting for sex (cohort). We acknowledge that the structure of the cohorts and sample size constraints in subgroup analyses may affect interpretability of our results. Sensitivity analyses pooling PWH and PWoH generally did not yield statistically significant interactions between HIV serostatus and pollution exposures. However, these unstratified data must be interpreted with caution given the differences between the PWH and PWoH groups that challenge direct comparison [51], and the lack of adjustment for HIV-related covariates in the pooled analyses. Similar to prior publications in the literature we draw conclusions from the analyses stratified by HIV status [41, 52]. 

Additionally, our analysis is limited by definition of diffusion impairment as < 80% predicted based on previously published work in the area [35], which may include some people who would be characterized as normal using lower limit of normal criteria, although this misclassification if present would only reduce our effect estimate. We did not further restrict DLCO to moderate impairment (< 60% predicted) due to small sample sizes.

Nevertheless, the strengths of our results include concordant findings in SGRQ and mMRC outcomes, as well as between COPD and impaired DLCO subgroups, supporting a potential pathophysiologic process for worse respiratory symptoms from PM_2.5_ air pollution in the setting of HIV and underlying chronic lung disease. In addition, our findings notably demonstrate an association between PM_2.5_ exposure and respiratory symptoms that is independent of tobacco smoking and degree of airflow obstruction in PWH with COPD.

## Conclusions

In summary, we demonstrated that PM_2.5_ pollution exposure was associated with respiratory morbidity in a sample of PWH with chronic lung disease. The combination of chronic HIV infection and air pollution may accelerate or activate lung disease. Further studies are needed to determine how chronic HIV infection predisposes individuals to respiratory symptom exacerbation and whether ambient air pollution plays a role in loss of lung function in PWH.

## Supplementary Information

Supplementary Material 1.

## Data Availability

Access to individual-level data from the MACS/WIHS Combined Cohort Study Data (MWCCS) may be obtained upon review and approval of a MWCCS concept sheet. Links and instructions for online concept sheet submission are on [the study website](https:/statepi.jhsph.edu/mwccs/work-with-us).

## Abbreviations

CI: Confidence interval
CMAQ: Community Multiscale Air Quality Modeling System
COPD: Chronic obstructive pulmonary disease
DLCO: Diffusing capacity of the lungs for carbon monoxide
FEV_1_: Forced expiratory volume in 1 second
FVC: Forced vital capacity
HIV: Human immunodeficiency virus
MACS: Multicenter AIDS Cohort Study
mMRC: Modified Medical Research Council dyspnea scale score
MWCCS: MACS (Multicenter AIDS Cohort Study) and WIHS (Women’s Interagency HIV Study) Combined Cohort Study
O_3_: Ozone
PFT: Pulmonary function test
PM_2.5_: Fine particulate matter with diameter < 2.5 μm
PWH: People with HIV
PWoH: People without HIV
SGRQ: St. George’s Respiratory Questionnaire
WIHS: Women’s Interagency HIV Study
