# Correction to: Greater ambient air pollution exposure is associated with worse respiratory symptoms in men and women with HIV and chronic lung disease: a cohort study

**DOI:** 10.1186/s12931-026-03576-8

**Published:** 2026-02-24

**Authors:** May-Lin Wilgus, Andrew Edmonds, Kayleigh P. Keller, Jessica L. Elf, Alison G. Abraham, Surya P. Bhatt, Russell G. Buhr, Mardge H. Cohen, Roger Detels, M. Bradley Drummond, Margaret A. Fischl, Robert Foronjy, H. Dean Hosgood, Laurence Huang, Ken M. Kunisaki, Meredith McCormack, Alison Morris, Sarath Raju, Catalina Ramirez, Jacqueline Rudolph, Valentina Stosor, Donald P. Tashkin, Adam Visconti, Sheri D. Weiser, Gina Wingood, Igor Barjaktarevic

**Affiliations:** 1 University of California Los Angeles, Los Angeles, CA 90095 USA; 2 University of North Carolina at Chapel Hill, Chapel Hill, NC USA; 3 Colorado State University, Fort Collins, CO USA; 4 University of Colorado, Aurora, CO USA; 5 University of Alabama at Birmingham, Birmingham, AL USA; 6 Stroger Hospital of Cook County Health and Human Services, Chicago, IL USA; 7 University of Miami School of Medicine, Miami, FL USA; 8 SUNY Downstate Health Sciences University, Brooklyn, NY USA; 9 Albert Einstein College of Medicine, Bronx, NY USA; 10 University of California San Francisco, San Francisco, CA USA; 11 Boise VA Medical Center, Boise, ID USA; 12 Johns Hopkins University, Baltimore, MD USA; 13 University of Pittsburgh School of Medicine, Pittsburgh, PA USA; 14 Center for Advanced Biotechnology and Medicine, Rutgers Robert Wood Johnson Medical School, Piscataway, NJ USA; 15 Northwestern University Feinberg School of Medicine, Chicago, IL USA; 16 Georgetown University, Washington, DC USA; 17 Columbia University, New York, NY USA

**Correction to: Respir Res 26, 349 (2025)**

**https://doi.org/10.1186/s12931-025–03398-0**

In the original publication of this article [1], the author’s name Laurence Huang was incorrectly written as Lawrence Huang. The original article has been corrected.

## References

[1] Wilgus ML, Edmonds A, Keller KP, et al. Greater ambient air pollution exposure is associated with worse respiratory symptoms in men and women with HIV and chronic lung disease: a cohort study. Respir Res. 2025;26:349. 41420180 10.1186/s12931-025-03398-0 PMC12717705

