# Supplementary material for: Greater ambient air pollution exposure is associated with worse respiratory symptoms in men and women with HIV and chronic lung disease: a cohort study
Source: Respir Res. 2025 Dec 19;26:349. doi: 10.1186/s12931-025-03398-0 (PMC12717705; doi:10.1186/s12931-025-03398-0)
Supplement: Supplementary file 1 — Supplementary Material 1. [file 12931_2025_3398_MOESM1_ESM.docx]

**Supplementary Table 1:** Air pollution by study site

| **Study site** | **O_3_ ppb** | | **PM_2.5_ µg/m^3^** | |
| --- | --- | --- | --- | --- |
|  | median (IQR) | mean (SD) | median (IQR) | mean (SD) |
| WIHS |  |  |  |  |
| Bronx, NY | 35.4 (35.0–35.9) | 35.5 (0.7) | 8.8 (8.7–8.9) | 8.7 (0.3) |
| Brooklyn, NY | 33.9 (33.6–34.1) | 34.0 (0.7) | 8.8 (8.6–9.0) | 8.8 (0.3) |
| Washington, DC | 35.4 (34.9–36.1) | 35.7 (1.7) | 8.5 (8.3–9.0) | 8.5 (0.6) |
| San Francisco, CA | 29.3 (27.8–31.6) | 30.8 (4.5) | 11.3 (10.9–11.9) | 11.5 (1.2) |
| Chicago, IL | 37.3 (36.8–37.7) | 37.3 (0.9) | 9.5 (9.3–9.7) | 9.5 (0.4) |
| Chapel Hill, NC | 39.2 (38.9–39.7) | 39.3 (0.7) | 8.6 (8.2–8.8) | 8.4 (0.6) |
| Atlanta, GA | 38.0 (37.7–38.5) | 38.0 (0.6) | 10.0 (9.8–10.2) | 10.0 (0.3) |
| Miami, FL | 34.3 (33.9–34.5) | 34.2 (0.5) | 8.1 (8.1–8.2) | 8.1 (0.2) |
| Birmingham, AL | 38.0 (37.5–38.3) | 37.9 (0.6) | 10.4 (10.1–10.6) | 10.3 (0.4) |
| Jackson, MS | 36.8 (36.4–37.1) | 36.6 (0.7) | 9.9 (9.6–10.2) | 9.8 (0.5) |
| MACS |  |  |  |  |
| Baltimore, MD | 38.6 (37.9–39.8) | 38.7 (1.3) | 8.3 (8.1–8.6) | 8.3 (0.4) |
| Chicago, IL | 36.3 (36.0–36.6) | 36.3 (1.0) | 9.4 (9.1–9.5) | 9.3 (0.5) |
| Pittsburgh, PA | 39.4 (39.2–39.6) | 39.2 (0.8) | 10.3 (9.8–10.4) | 10.1 (0.7) |
| Los Angeles, CA | 43.7 (42.2–45.1) | 43.9 (3.7) | 11.3 (11.0–11.5) | 11.0 (1.3) |

MACS Multicenter AIDS Cohort Study, WIHS Women’s Interagency HIV Study, IQR interquartile range, SD standard deviation, PM_2.5_ particulate matter <2.5 microns, O_3_ ozone, ppb parts per billion

**Supplementary Table 2:** Relationships between PM_2.5_ and O_3_ exposures and lung function in MACS and WIHS: PWH and PWoH pooled, including interaction term between HIV serostatus and pollution exposure

|  | | **MACS**  N=338 (PWoH 192, PWH 146)  Effect estimate^a^ (95% CI) | | **WIHS**  N=1073 (PWoH 304, PWH 769)  Effect estimate^a^ (95% CI) | |
| --- | --- | --- | --- | --- | --- |
|  |  | **Adjusted**^b^ | **P-value**^c^ | **Adjusted**^b^ | **P-value**^c^ |
| PM_2.5_  1 µg/m^3^  increase | FEV_1_/FVC  *PWoH*  *PWH* | -0.004 (-0.01, 0.003)  0.006 (-0.003, 0.02) | 0.07 | **-0.01 (-0.02, -0.003)**  0.0009 (-0.004, 0.006) | 0.01 |
|  | % predicted FEV_1_  *PWoH*  *PWH* | 0.41 (-1.36, 2.18)  **2.31 (0.05, 4.57)** | 0.2 | -0.05 (-1.95, 1.85)  0.52 (-0.6, 1.64) | 0.6 |
|  | % predicted FVC  *PWoH*  *PWH* | 0.89 (-0.66, 2.44)  1.57 (-0.42, 3.55) | 0.6 | 1.43 (-0.36, 3.22)  0.38 (-0.68, 1.44) | 0.3 |
|  | % predicted DLCO  *PWoH*  *PWH* | 0.7 (-0.8, 2.21)  1.76 (-0.29, 3.81) | 0.4 | 0.23 (-1.8, 2.25)  1.1 (-0.11, 2.26) | 0.5 |
| O_3_  3 ppb  increase | FEV_1_/FVC  *PWoH*  *PWH* | 0.0003 (-0.008, 0.009)  **0.01 (0.003, 0.02)** | 0.06 | -0.003 (-0.01, 0.005)  -0.004 (-0.01, 0.002) | 0.9 |
|  | % predicted FEV_1_  *PWoH*  *PWH* | 0.67 (-1.46, 2.8)  1.66 (-0.75, 4.07) | 0.5 | -0.91 (-2.82, 0.99)  -0.08 (-1.42, 1.25) | 0.5 |
|  | % predicted FVC  *PWoH*  *PWH* | 0.62 (-1.25, 2.5)  0.21 (-1.91, 2.32) | 0.8 | -0.48 (-2.28, 1.32)  0.34 (-0.92, 1.61) | 0.5 |
|  | % predicted DLCO  *PWoH*  *PWH* | **1.91 (0.13, 3.7)**  1.5 (-0.72, 3.73) | 0.8 | -0.74 (-2.62, 1.14)  -0.5 (-1.85, 0.85) | 0.8 |

^a^ Difference in lung function for 1 µg/m^3^ increase in PM_2.5_ exposure or 3 ppb increase in O_3_ exposure

^b^ Separate models for MACS and WIHS, adjusted for HIV serostatus, age, race, ethnicity, current smoking, smoking pack-years, and BMI

^c^ P-value for interaction term between HIV serostatus and pollution exposure

MACS Multicenter AIDS Cohort Study, WIHS Women’s Interagency HIV Study, PWH people with HIV, PWoH people without HIV, CI confidence interval, FVC forced vital capacity, FEV_1_ forced expiratory volume at 1 second, DLCO diffusion capacity for carbon monoxide, PM_2.5_ particulate matter <2.5 microns, O_3_ ozone, ppb parts per billion; boldface entries indicate a statistically significant correlation

**Supplementary Table 3:** Relationships between PM_2.5_ and O_3_ exposures and respiratory symptom scores in MACS and WIHS: PWH and PWoH pooled, including interaction term between HIV serostatus and pollution exposure

|  | | **MACS**  N=338 (PWoH 192, PWH 146)  Effect estimate^a^ (95% CI) | | **WIHS**  N=1073 (PWoH 304, PWH 769)  Effect estimate^a^ (95% CI) | |
| --- | --- | --- | --- | --- | --- |
|  |  | **Adjusted**^b^ | **P-value**^c^ | **Adjusted**^b^ | **P-value**^c^ |
| PM_2.5_  1 µg/m^3^  increase | SGRQ  *PWoH*  *PWH* | 0.2 (-1.05, 1.44)  0.34 (-1.24, 1.93) | 0.8 | 1.17 (-0.41, 2.74)  0.72 (-0.21, 1.66) | 0.6 |
|  | mMRC  *PWoH*  *PWH* | -0.007 (-0.09, 0.08)  -0.02 (-0.12, 0.09) | 0.9 | - 1. (-0.07, 0.18)   0.05 (-0.02, 0.13) | 0.9 |
|  | mMRC binary^d^  *PWoH*  *PWH* | 0.78 (0.42, 1.44)  0.98 (0.57, 1.71) | 0.6 | **1.28 (1.01, 1.63)**  1.07 (0.94, 1.23) | 0.2 |
| O_3_  3 ppb  increase | SGRQ  *PWoH*  *PWH* | -0.45 (-1.98, 1.08)  -0.32 (-2.01, 1.37) | 0.9 | **-1.69 (-3.28, -0.11)**  -0.05 (-1.17, 1.06) | 0.09 |
|  | mMRC  *PWoH*  *PWH* | -0.006 (-0.1, 0.09)  0.03 (-0.08, 0.14) | 0.7 | -0.03 (-0.16, 0.1)  0.04 (-0.05, 0.13) | 0.4 |
|  | mMRC binary^d^  *PWoH*  *PWH* | 0.72 (0.32, 1.6)  1.24 (0.68, 2.24) | 0.3 | 0.89 (0.7, 1.13)  1.07 (0.91, 1.26) | 0.2 |

^a^ Difference in SGRQ and mMRC for 1 µg/m^3^ increase in PM_2.5_ exposure or 3 ppb increase in O_3_ exposure

^b^ Separate models for MACS and WIHS, adjusted for HIV serostatus, age, race, ethnicity, current smoking, smoking pack-years, and BMI

^c^ P-value for interaction term between HIV serostatus and pollution exposure

^d^ Binary mMRC outcome: high (2-4) vs. low (0-1), estimate is odds ratio from logistic regression

MACS Multicenter AIDS Cohort Study, WIHS Women’s Interagency HIV Study, PWH people with HIV, PWoH people without HIV, CI confidence interval, SGRQ St. George’s Respiratory Questionnaire score, mMRC Modified Medical Research Council dyspnea scale score, PM_2.5_ particulate matter <2.5 microns, O_3_ ozone, ppb parts per billion; boldface entries indicate a statistically significant correlation

**Supplementary Table 4:** Relationships between PM_2.5_ and O_3_ exposures and respiratory symptom scores in participants with COPD and impaired diffusion capacity: PWH and PWoH pooled, including interaction term between HIV serostatus and pollution exposure

|  | | **MACS/WIHS COPD Subgroup**  N=140 (PWoH 96, PWH 44)  Effect estimate^a^ (95% CI) | | **MACS/WIHS Impaired Diffusion Capacity Subgroup**  N=240 (PWoH 91, PWH 149)  Effect estimate^a^ (95% CI) | |
| --- | --- | --- | --- | --- | --- |
|  |  | **Adjusted**^b^ | **P-value**^c^ | **Adjusted**^b^ | **P-value**^c^ |
| PM_2.5_  1 µg/m^3^  increase | SGRQ  *PWoH*  *PWH* | 2.77 (-0.97, 6.5)  3.42 (-0.01, 6.85) | 0.8 | -0.5 (-3, 2.04)  **2.22 (0.4, 4.04)** | 0.09 |
|  | mMRC  *PWoH*  *PWH* | 0.02 (-0.25, 0.29)  0.19 (-0.06, 0.44) | 0.4 | -0.03 (-0.2, 0.14)  **0.16 (0.03, 0.28)** | 0.08 |
|  | mMRC binary^d^  *PWoH*  *PWH* | 1.25 (0.74, 2.11)  1.35 (0.84, 2.19) | 0.8 | 0.99 (0.64, 1.53)  **1.4 (1.03, 1.9)** | 0.2 |
| O_3_  3 ppb  increase | SGRQ  *PWoH*  *PWH* | -3.22 (-8.19, 1.75)  -0.77 (-4.54, 3) | 0.4 | **-3.36 (-6.36, -0.35)**  -0.27 (-2.68, 2.15) | 0.1 |
|  | mMRC  *PWoH*  *PWH* | 0.16 (-0.2, 0.51)  -0.05 (-0.32, 0.22) | 0.3 | -0.15 (-0.36, 0.06)  0.004 (-0.16, 0.17) | 0.2 |
|  | mMRC binary^d^  *PWoH*  *PWH* | 1.43 (0.7, 2.9)  0.78 (0.44, 1.37) | 0.2 | 0.71 (0.41, 1.21)  1.03 (0.7, 1.5) | 0.2 |

^a^ Difference in SGRQ and mMRC for 1 µg/m^3^ increase in PM_2.5_ exposure or 3 ppb increase in O_3_ exposure

^b^ Separate models for MACS and WIHS, adjusted for HIV serostatus, sex, age, race, ethnicity, current smoking, smoking pack-years, and BMI

^c^ P-value for interaction term between HIV serostatus and pollution exposure

^d^ Binary mMRC outcome: high (2-4) vs. low (0-1), estimate is odds ratio from logistic regression

MACS Multicenter AIDS Cohort Study, WIHS Women’s Interagency HIV Study, PWH people with HIV, PWoH people without HIV, CI confidence interval, SGRQ St. George’s Respiratory Questionnaire score, mMRC Modified Medical Research Council dyspnea scale score, FEV_1_ forced expiratory volume at 1 second, PM_2.5_ particulate matter <2.5 microns, O_3_ ozone, ppb parts per billion; boldface entries indicate a statistically significant correlation
